# Risk factors for bat contact and consumption behaviors in Thailand; a quantitative study

**DOI:** 10.1186/s12889-020-08968-z

**Published:** 2020-06-03

**Authors:** Kanokwan Suwannarong, Sutin Chanabun, Phitsanuruk Kanthawee, Santisith Khiewkhern, Paisit Boonyakawee, Kangsadal Suwannarong, Chutarat Saengkul, Nisachon Bubpa, Alongkorn Amonsin

**Affiliations:** 1 Center of Excellence for Emerging and Re-emerging Infectious Diseases in Animals, Chulalongkorn University, Bangkok, Thailand; 2 Sirinthorn College of Public Health Khon Kaen, Ministry of Public Health, Khon Kaen, Thailand; 3 School of Health Science, Mae Fah Luang University, Chiang Rai, Thailand; 4 Faculty of Public Health, Mahasarakham University, Mahasarakham, Thailand; 5 Sirinthorn College of Public Health Trang, Ministry of Public Health, Trang, Thailand; 6 The Office of Disease Prevention and Control 7 Khon Kaen, Khon Kaen, Thailand; 7 Faculty of Public Health, Nakhon Sawan Campus, Mahidol University, Nakhon Sawan, Thailand; 8 Faculty of Nursing, Khon Kaen University, Khon Kaen, Thailand; 9 Department of Veterinary Public Health, Faculty of Veterinary Science, Chulalongkorn University, Bangkok, 10330 Thailand

**Keywords:** Bat, Behavior, Contact, Consumption, Risk factors, Thailand

## Abstract

**Background:**

Bats serve as an important reservoir for emerging infectious diseases. Bat contact and consumption, which persists in Asia, poses risks for the transmission of bat-borne infections.

**Methods:**

An analytical cross-sectional survey for risk factors associated with bat contact and consumption behaviors was conducted in ten provinces of Thailand from May 2016 to December 2017. A standardized questionnaire administered through face-to-face interviews was used to collect information from 626 villagers who lived in or nearby areas of high bat density. The questionnaire contained 23 independent variables related to sociodemographic, knowledge, attitudes, practices, and perceptions.

**Results:**

The respondents (*n* = 626) were 285 females and 341 males, mean age of respondents was 47.58 years-old and lived in rural setting. Our results showed that 36.42% of respondents (n_1_ = 228) in 10 provinces reported bat contact during the past 6 months. Furthermore, 15.34% of respondents (n_2_ = 96) in 9 out of 10 provinces reported of having consumed bat meat in the past 6 months. Risk factors for bat contact included sex (male) (OR = 1.56, 95% CI 1.09–2.28), educational attainment (lower than secondary school) (OR = 1.45, 95% CI 1.02–2.18), and the consideration of bats as being economically beneficial to the community (OR = 3.18, 95% CI 2.03–4.97), while agriculture-related occupation (OR = 0.54, 95% CI 0.37–0.79), knowledge that it is safe to eat bats (OR = 0.58, 95% CI 0.37–0.93), practice of allowing children to play with bats (OR = 0.65, 95% CI 0.44–0.96), and attitude of feeling safe in areas where bats live (OR = 0.56, 95% CI 0.38–0.86) were statistically significant protective factors against bat contact. Risk factors for bat consumption included sex (male) (OR = 2.48, 95% CI 1.49–4.11) and educational attainment (lower than secondary school) (OR = 2.21, 95% CI 1.27–3.85), while knowledge of whether bats are safe to eat (OR = 0.04, 95% CI 0.01–0.25), knowledge of whether there are laws pertaining to hunting bats for consumption (OR = 0.35, 95% CI 0.18–0.71), and the practice of allowing children to play with bats (OR = 0.51, 95% CI 0.31–0.81) were statistically significant protective factors against bat consumption.

**Conclusions:**

This study provides a better understanding of the sociodemographic factors, knowledge, attitudes, perceptions and practices that might influence bat contact and bat consumption behaviors. Information on risk factors can be used for the development of appropriate education and communication interventions to promote proper knowledge, attitudes and practices regarding bats and bat-borne zoonotic diseases in Thailand and other areas in the Southeast Asia region with similar environmental and cultural characteristics.

## Background

The human and wildlife interface has been a global concern in the past decade due to several zoonotic disease outbreaks related to wildlife contact. Pathogen transmission may occur through several routes, e.g., inhalation [1]; bites [2–4]; scratches [5]; hunting [6–9]; guano use as fertilizer [10]; food consumption [6, 11, 12]; handling, slaughtering and butchering; drinking water or food contaminated with saliva or feces [13]; and possible human-to-human transmission.

Bats are important reservoirs of several zoonotic pathogens. Bat-borne diseases in humans have been reported worldwide and have been considered global concerns; these include the Nipah virus [13, 14], the Hendra virus [15], Ebola [16], lyssaviruses [17], and severe acute respiratory syndrome coronavirus (SARS-CoV) [2, 18, 19]. It has been documented that several factors influence human contact with bats. For example, previous studies in Asia, and North America reported that sex [6], occupation [4], and locations of bat habitats [6] influence human-bat interactions. Bats play a cultural role in Thailand including medicinal, religious, and culinary [10]. A better understanding of human-bat interactions is critical to understanding zoonotic disease spillover and has been insufficiently studied [20]. Especially in Thailand, the understanding of human-bat interactions and the epidemiological links involved in bat-borne disease and bat consumption is still insufficient.

The objective of this study was to determine the factors associated with bat contact and bat consumption behaviors in ten provinces of Thailand from May 2016 to December 2017. Our results will support the development of appropriate education and communication interventions to promote proper knowledge, attitudes and practices regarding bats and bat-borne zoonotic diseases in Thailand and Southeast Asia.

## Methods

### Study design

An analytical cross-sectional study was conducted to identify the factors associated with bat contact and bat consumption behaviors among persons who lived in or near areas of high bat density within at least 6 months before the study. The questionnaire interviews were conducted from May 2016 to July 2017. The Chulalongkorn University Institution Review Boards (IRBs) and Chiang Rai provincial health office approved the human study (Ref No. 034/59 and 26/ 2559). This study obtained agreements from local administrative offices and the chiefs of villages to conduct data collection at the study sites. Written informed consent forms were signed by the participants after they received information about the objectives of the study and prior to the interviews.

### Study sites and study population selection

Different sampling methods were applied to select the study sites and study populations. The whole country was first stratified into four regions; central, northern, northeastern and southern Thailand. Provinces, districts and villages in each region were then purposively selected based on; 1) a high density of bats in the villages/study areas, 2) a potential bat-human interface was observed by researchers during scoping visits, discussed with some villagers and/or local authorities, and 3) information obtained from the relevant local and national authorities such as the Ministry of Natural Resources and Environment (MNRE), and the Ministry of Public Health (MoPH). Lastly, a simple random sampling method (SRS) was used to select respondents from official household registry records that were obtained from the local health promotional hospitals in the villages.

**Fig. 1:**
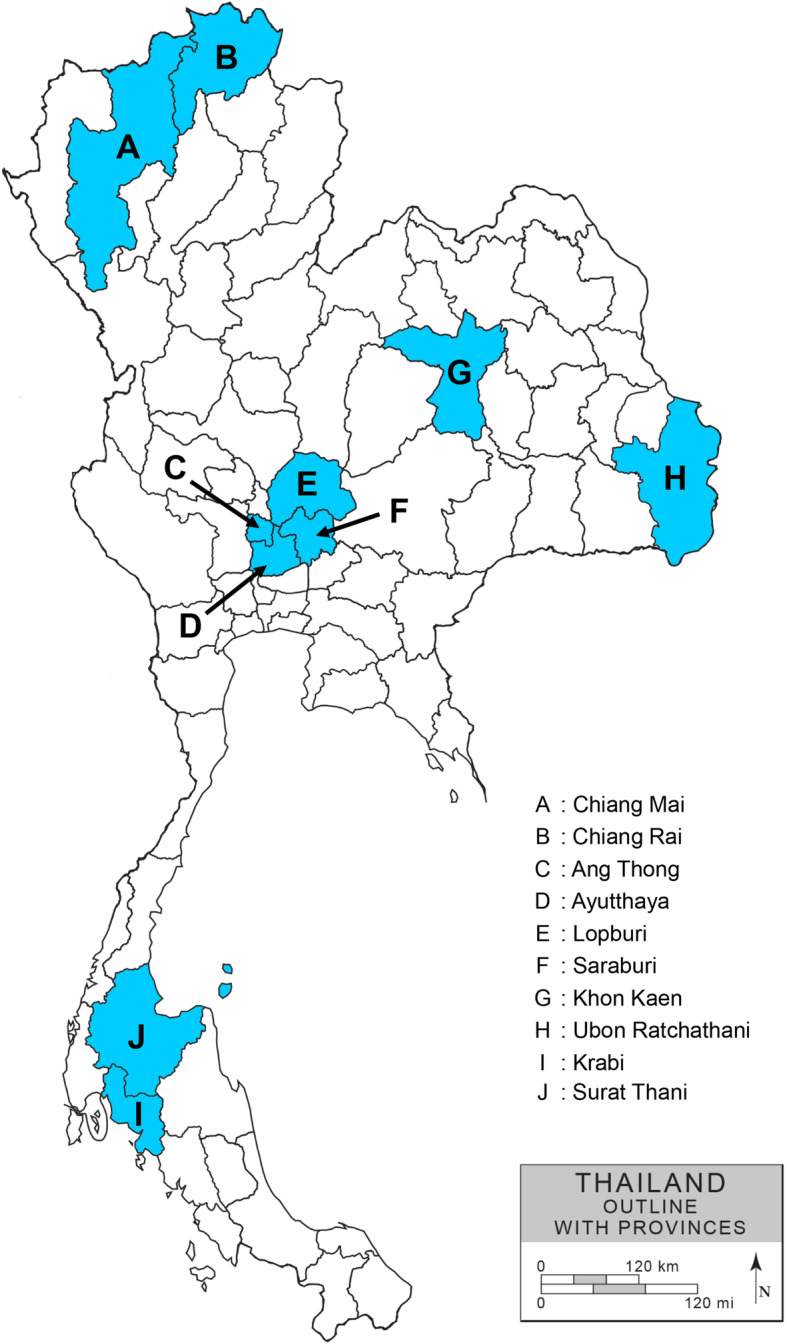
Maps of provinces and study sites in Thailand (Thailand map is purchased with permission of© Copyright 2007 by World Trade Press. All Rights Reserved)

In this study, 10 provinces were selected as representatives of regions in Thailand that have different living characteristics and practices. The provinces were Ang Thong, Ayutthaya, Lopburi and Saraburi (central provinces); Chiang Mai and Chiang Rai (northern provinces); KhonKaen and UbonRatchathani (northeastern provinces); and Krabi and Surat Thani (southern provinces) (Fig. 1 and Table 1). Bats from each study site were collected for species identification using physical characteristics and DNA sequence variations in mitochondrial cytochrome-b (CytB) [21].

**Table 1:** Numbers of districts, villages, respondents (*N* = 626), numbers of respondents who reported bat contact (*n* = 228), and number of respondents who reported bat consumption (*n* = 96)

				No. of respondents (%)		
Regions	Provinces	No. of districts	No. of villages	No. of respondents	Reported bat contact	Reported bat consumption
Northern	Chiang Mai	1	4	80 (12.78%)	28 (12.28%)	27 (28.13%)
Northern	Chiang Rai	1	2	60 (9.58%)	20 (8.77%)	13 (13.54%)
Central	Ang Thong	1	1	64 (10.22%)	26 (11.40%)	0 (0.00%)
Central	Ayutthaya	1	1	60 (9.58%)	24 (10.53%)	1 (1.04%)
Central	Lopburi	3	4	77 (33.77%)	25 (10.96%)	1 (1.04%)
Central	Saraburi	1	1	42 (6.71%)	21 (9.21%)	3 (3.13%)
Northeastern	Khon Kaen	4	8	69 (11.02%)	21 (9.21%)	3 (3.13%)
Northeastern	Ubon Ratchathani	1	1	60 (9.58%)	24 (10.53%)	18 (18.75%)
Southern	Krabi	1	2	60 (9.58%)	19 (8.33%)	14 (14.58%)
Southern	Surat Thani	5	13	54 (8.63%)	20 (8.77%)	16 (16.67%)
		19	37	626 (100%)	228 (100%)	96 (100%)

### Sample size estimation, respondents, and recruitment procedures

The inclusion criteria for respondents were males or females between 20 and 75 years of age who had lived in the selected areas for at least 6 months before data collection and were willing to participate in this study. The sample size calculation for this study was as follows:

$$ \mathrm{n}=\frac{\left[{Z}^2\left(1-\alpha /2\right)\ p\ \left(1-p\right)\right]}{{\mathrm{d}}^2} $$

Here, *p* = proportion of participants with bat contact. Based on a previous study, 23.00% of respondents reported bat consumption at some time in their lives [22]. The variable p is 0.23 for this calculation, z = 1.96 (95% confidence interval), and d (margin of error) = 5%. Therefore, the calculated sample size was 340. To increase the power, the sample size was elevated to 626.

Simple random sampling (SRS) was used on the lists of the respondents retrieved from health promotional hospitals in the study areas. After SRS of respondents was conducted, the participants were contacted by trained researchers for data collection. Using a standardized questionnaire, a face-to-face interview was conducted with the respondents in a place that was not too secluded but still free from disturbances.

### A standardized questionnaire

A questionnaire was designed to collect information on bat contact and bat consumption behaviors. This questionnaire was modified from previous reports [10, 23, 24]. The questionnaire interview was administered to collect information on sociodemographic factors, knowledge, attitudes, practices and perceptions for bat contact and bat consumption behaviors. The questionnaire was pretested with 30 respondents in a district with the same environmental and population characteristics as the actual selected sites. The questionnaire was refined per the pretest results. Field researchers were trained in its administration and in conducting questionnaire interviews with written informed consent forms obtained prior to the interviews. After the interviews, the information was checked for validity and precision before it was entered into SPSS Software Version 22 (Chicago, IL).

### Study variables for risk factors for bat contact and bat consumption behaviors

Independent variables (*n* = 23) were included in the analysis. The variables were based on previous relevant studies [10, 25–29]. The independent variables, including the sociodemographic information, knowledge, attitudes, practices, and perceptions were as follows:

- Sociodemographic information (10 variables)

- Sex (male vs female)
- Age group (> age of 36 years vs ≤ 36 years)
- Occupation (farmer vs other)
- Marital status (married and cohabiting vs single)
- Family size (≥ 2 people vs < 2 people)
- Family monthly income (≤ 15,000 Baht vs > 15,000)
- Own a car (yes vs no)
- Own a motorcycle (yes vs no)
- Number of children (none vs > 1 child)
- Education attainment (< secondary school vs other)

b) Knowledge, attitudes, practices and perceptions (13 variables)

- Humans can get diseases from bats (true vs false)
- There are no concerns about getting diseases from bats (true vs false)
- Bats can transmit diseases to humans (true vs false)
- Bats are economically beneficial to the community (agree vs disagree)
- One can contract diseases if exposed to bats (true vs false)
- One can contract diseases by drinking water from the same places as bats (true vs false)
- One can contract diseases by eating fruits left by bats (true vs false)
- Bat guano is safe to use (true vs false)
- It is safe to eat bats (true vs false)
- There are laws related to hunting bats for consumption (true vs false)
- Children are allowed to play with bats (true vs false)
- Dead bats that are found are brought home for food (true vs false)
- One feels safe in areas where bats live (true vs false)

The dependent variable was a report of either one of bat contact experiences by the study respondents. List of bat contact experiences included the following:

- Found dead bat(s) in house
- Found live bat(s) in house, the community or tourist location
- Cleaned bat guano from house or the community
- Cleaned bat carcasses from house or the community
- Bat guano mining/collecting
- Use of bat guano
- Bitten by a bat
- Consumed bats for food
- Other activities, e.g., hunted bats, exposed to urine of bats

### Analysis of risk factors for bat contact and bat consumption behaviors

The interviewed data were entered into and analyzed by SPSS software version 22 (Chicago, IL). After data cleaning, the dependent and independent variables were assessed. Data were analyzed in 2 steps. First, bivariate analysis was performed, in which the degree of association of each variable was computed and each of the independent variables was analyzed separately. Second, a stepwise logistic regression model was constructed including independent variables that had *p* values ≤0.15 results in bivariate analysis. This second step used *p* ≤ 0.05 as the cutoff point for identifying statistically significant variables.

## Results

### Sociodemographic information of respondents

In this study, 626 respondents participated in the questionnaire interview. These respondents from 37 villages of 16 districts in 10 provinces in Thailand (Fig. 1 and Table 1). Bats species from each study site were identified: Chiang Mai *(Scotophilus heathii* and *Megaderma spasma)*, Chiangxs Rai *(Taphozous melanopogon)*, Lopburi (*Taphozous melanopogon* and *Chaerephon plicatus)*, Khon Kaen (*Chaerephon plicatus)*, and Ayutthaya, Ang Thong, Saraburi (*Pteropus lylei*). Of the 626 respondents who participated in the questionnaire interview, 228 (36.42%) respondents from 10 provinces reported having experienced bat contact during the past 6 months before data collection. Moreover, 96 (15.34%) respondents in 9 out of 10 provinces reported having consuming bat meat in the past 6 months.

The 626 respondents were 285 females and 341 males. The mean age of the respondents was 47.58 years. Approximately 21.09% of respondents were aged > 36 years. Most of them lived in rural settings (93.29%) and were married or cohabiting (84.03%). Respondents worked as farmers (rice, grains or vegetables) (35.78%), followed by temporary employees (22.68%), government officers (10.54%), housewives (7.03%), and shop vendors/owners (5.59%). Most of the respondents (65.65%) had attained educational levels lower than secondary school, and 80.35% had families composed of more than 2 persons. Most respondents had a monthly family income of ≤15,000 Baht (500 USD) (69.97%). Most respondents owned a motorcycle (91.69%), while fewer owned a car (50.16%).

### Bat contact experiences among respondents

In this study, 228 respondents in 10 provinces reported a bat contact experience during the past 6 months before data collection. Among those 228 respondents, 56.14% reported encountering live bats in a house, the community or a tourist location, while 42.10% reported eating bats for food, 34.21% found bat guano in a house or the community, 28.51% found dead bats in the house, 20.18% participated in bat guano mining/collecting, 19.74% cleaned bat carcasses from a house or the community, 10.96% used bat guano as fertilizer, 17.98% were involved in other contact activities (e.g., took bats from nets), and 7.02% been bitten by a bat in the past 6 months (Table 2).

**Table 2:** Details of bat contact experiences among 228 respondents who reported bat contact experiences (dependent variables)

Bat contact experience	No. of respondents who reported bat contact (%)^a^
Found dead bat(s) in house	65 (28.51)
Found live bat(s) in house, community or tourist location	128 (56.14)
Cleaned bat guano from house or the community	78 (34.21)
Cleaned bat carcasses from house or the community	45 (19.74)
Bat guano mining/ collecting	46 (20.18)
Used bat guano	25 (10.96)
Been bitten by a bat	16 (7.02)
Consumed bats for food	96 (42.10)
Other activities e.g. hunted bat(s), exposed to urine of bat(s)	41 (17.98)

^a^multiple responses

### Knowledge, attitudes, practices and perceptions regarding bat contact experiences

In this study, the questionnaire interview contained 10 sociodemographic questions and 13 questions related to the knowledge, attitudes, practices and perceptions regarding bat contact experiences. Bivariate analysis was performed to determine the associations between bat contact experiences and twenty-three independent variables. Of the 23 independent variables, 11 variables had significantly associations in the bivariate analysis, using *p* ≤ 0.15 as a cutoff point: sex (male), age group (> 36 years), occupation (agriculture-related occupation), educational attainment (< secondary school), family monthly income (< 15,000 Baht), no concerns about getting diseases from bats, considered bats to be economically beneficial to the community, believed it is safe to eat bats, allowed children to play with bats, and felt safe in areas where bats live (Table 3). All 11 variables were included in the stepwise logistic regression analysis for bat contact experiences. The results showed that sex (male) (OR = 1.56, 95% CI 1.09–2.28, *p* = 0.014), educational attainment (< secondary school) (OR = 1.45, 95% CI 1.02–2.18, *p* = 0.041), and considered bats to be economically beneficial to the community (OR = 3.18, 95% CI 2.03–4.97, *p* < 0.001) were statistically significant associated with bat contact experiences, while occupation (agriculture-related occupation) (OR = 0.54, 95% CI 0.37–0.79, *p* = 0.002), believed it is safe to eat bats (OR = 0.58, 95% CI 0.37–0.93, *p* = 0.023), allowed children to play with bats (OR = 0.65, 95% CI 0.44–0.96, *p* = 0.031), and felt safe in areas where bats live (OR = 0.56, 95% CI 0.38–0.86, *p* = 0.007) were statistically significant protective factors against bat contact experiences (Table 4).

**Table 3:** Bivariate analysis of bat contact experiences (Counts (n_1_ = 228), odds ratios, confidence intervals and *p*-values of independent variables)

Variables	Total number (*N* = 626)	Reported bat contact (*n* = 228)		Unadjusted odds ratio	95%Cl	*P*-value
		n	%			
Sociodemographic information						
1. Sex						
*Male*	341	141	61.84	0.62	0.45–0.87	0.005*
*Female*	285	87	38.16	1		
2. Age group						
*> 36 years*	132	41	17.98	1.35	0.89–2.04	0.150*
≤ *36 years*	494	187	82.02	1		
3. Occupation						
*Agriculture-related occupation*	224	66	28.95	1.62	1.14–2.29	0.007*
*Other*	402	162	71.05	1		
4. Marital status						
*Married or cohabiting*	526	188	82.46	1.20	0.77–1.86	0.417
*Single*	100	40	17.54	1		
5. Family size						
*≤ 2 people*	123	40	17.54	1.24	0.82–1.88	0.316
> *2 people*	503	188	82.46	1		
6. Number of children						
*0 children*	195	74	32.46	0.91	0.64–1.29	0.593
> *1 children*	431	154	67.54	1		
7. Educational attainment						
*≤* Secondary school	411	159	69.74	0.75	0.53–1.06	0.104*
Other	215	69	30.26	1		
8. Family monthly income						
≤ *15,000 Baht*	438	150	65.79	1.36	0.96–1.94	0.084*
*> 15,000 Baht*	188	78	34.21	1		
9. Own a car						
*Yes*	314	110	48.25	1.13	0.81–1.56	0.468
*No*	312	118	51.75	1		
10. Own a motorcycle						
*Yes*	574	210	92.11	0.92	0.51–1.67	0.777
*No*	52	18	7.89	1		
Knowledge, attitudes, practices and perceptions						
11. Human can get diseases from bats						
*True*	40	15	6.58	0.95	0.49–1.85	0.884
*False*	586	213	93.42	1		
12. There are no concerns about getting diseases from bats						
*True*	48	24	10.53	0.64	0.43–0.95	0.026*
*False*	578	204	89.47	1		
13. Bats can transmit diseases to humans						
*True*	84	28	12.28	1.17	0.72–1.90	0.527
*False*	542	200	87.72			
14. Bats are economically beneficial to the community						
*Agree*	108	65	28.51	0.30	0.20–0.47	< 0.001*
*Disagree*	518	163	71.49	1		
15. One can contract diseases if exposed to bats						
*True*	101	37	16.63	0.99	0.64–1.54	0.961
*False*	525	191	83.77	1		
16. One can contract diseases by drinking water from the same place as bats do						
*True*	139	48	21.05	1.11	0.75–1.65	0.600
*False*	487	180	78.77	1		
17. One can contract diseases by eating fruit left by bats						
*True*	170	64	28.07	0.93	0.65–1.14	0.697
*False*	456	164	71.93	1		
18. Bat guano is safe to use						
*True*	58	14	6,14	1.90	1.02–3.55	0.041*
*False*	568	214	93.86			
19. Bats are safe to eat						
*True*	169	37	16.23	2.56	1.70–3.86	< 0.001*
*False*	457	191	83.77			
20. There are laws related to hunting bats for consumption						
*True*	196	66	28.95	1.19	0.84–1.70	0.335
*False*	430	162	71.05	1		
21. Children are allowed to play with bats						
*True*	431	138	60.53	1.82	1.29–2.57	0.001*
*False*	195	90	39.47	1		
22. Found dead bat(s) and brought home for food						
*True*	518	195	85.53	0.73	0.47–1.14	0.164
*False*	108	33	14.47	1		
23. Feel safe in areas where bats live						
*True*	210	47	20.61	2.67	1.83–3.90	< 0.001*
*False*	416	181	79.39	1		

^*^Cut-off point for stepwise logistic regression at *p* < 0.15

**Table 4:** Stepwise logistic regression analysis of bat contact experiences (Coefficient, odds ratios, confidence intervals and *p*-values of independent variables)

Variables	Coefficient	Odds ratio	95% CI	*P*-value
Sociodemographic information				
Sex (male)	0.45	1.56	1.09–2.28	0.014*
Occupation (agriculture-related occupation)	−0.61	0.54	0.37–0.79	0.002*
Educational attainment (< secondary school level)	0.39	1.45	1.02–2.18	0.041*
Knowledge, attitudes, practices and perceptions				
Considered bat(s) to be economically beneficial to the community (agreed)	1.16	3.18	2.03–4.97	< 0.001*
Bat are safe to eat (true)	−0.54	0.58	0.37–0.93	0.023*
Children are allowed to play with bats (true)	−0.43	0.65	0.44–0.96	0.031*
Felt safe in areas where bats live (true)	−0.58	0.56	0.38–0.86	0.007*

*Statistically significant at *p* < 0.05

### Bat consumption behaviors among respondents

Among the 626 respondents, 96 respondents (15.34%; 28 females and 68 males) reported eating bats in the past 6 months. Of the respondents who reported contacts with bats, 42.10% (96/228) reported eating bats in the past 6 months. They also reported that bats were hunted, butchered, slaughtered, and cooked by several categories of persons (e.g., husbands, wives, neighbors, children, or hunters) in their communities. In this study, 170 out of 626 respondents (27.16%) reported having eaten bats in their lifetime, of whom 62.35% (106/170) reported consuming bats more than 10 years ago, 11.18% (19/170) who reported eating bats during the last 1–10 years, 20.00% (34/170) who reported eating bats within the past 12 months, 2.94% (5/ 170) who reported eating bats in the past month, 2.35% (4/170) who reported eating bats within week of data collection, and 1.18% (2/170) who reported eating bats within the preceding week.

Of the 96 respondents who reported eating bats, 70.83% were male and 83.33% were < 36 years old (mean age of respondents 47.21 years old). Most respondents were married or cohabiting (85.42%) and had more than one child (69.79%). The occupations of respondents were farmers (rice, grains or vegetables) (39.58%), followed by temporary employees (29.17%). Most respondents had attained an educational level < secondary school (79.17%) and had families composed of more than 2 persons (76.04%). Most of the respondents (66.67%) had monthly family income < 15,000 Baht (500 USD). Some respondents owned a car (42.71%), majority owned a motorcycle (96.88%). Of the 96 respondents, those in the northern region, Chiang Mai (28.13%) and Chiang Rai (13.54%), reported more bat consumption than those in other provinces, including those in the northeastern region, Ubon Ratchathani (18.75%), southern region, Surat Thani (16.67%) and Krabi (14.58%). On the other hand, respondents in the central region reported less bat consumption (Table 1).

**Fig. 2:**
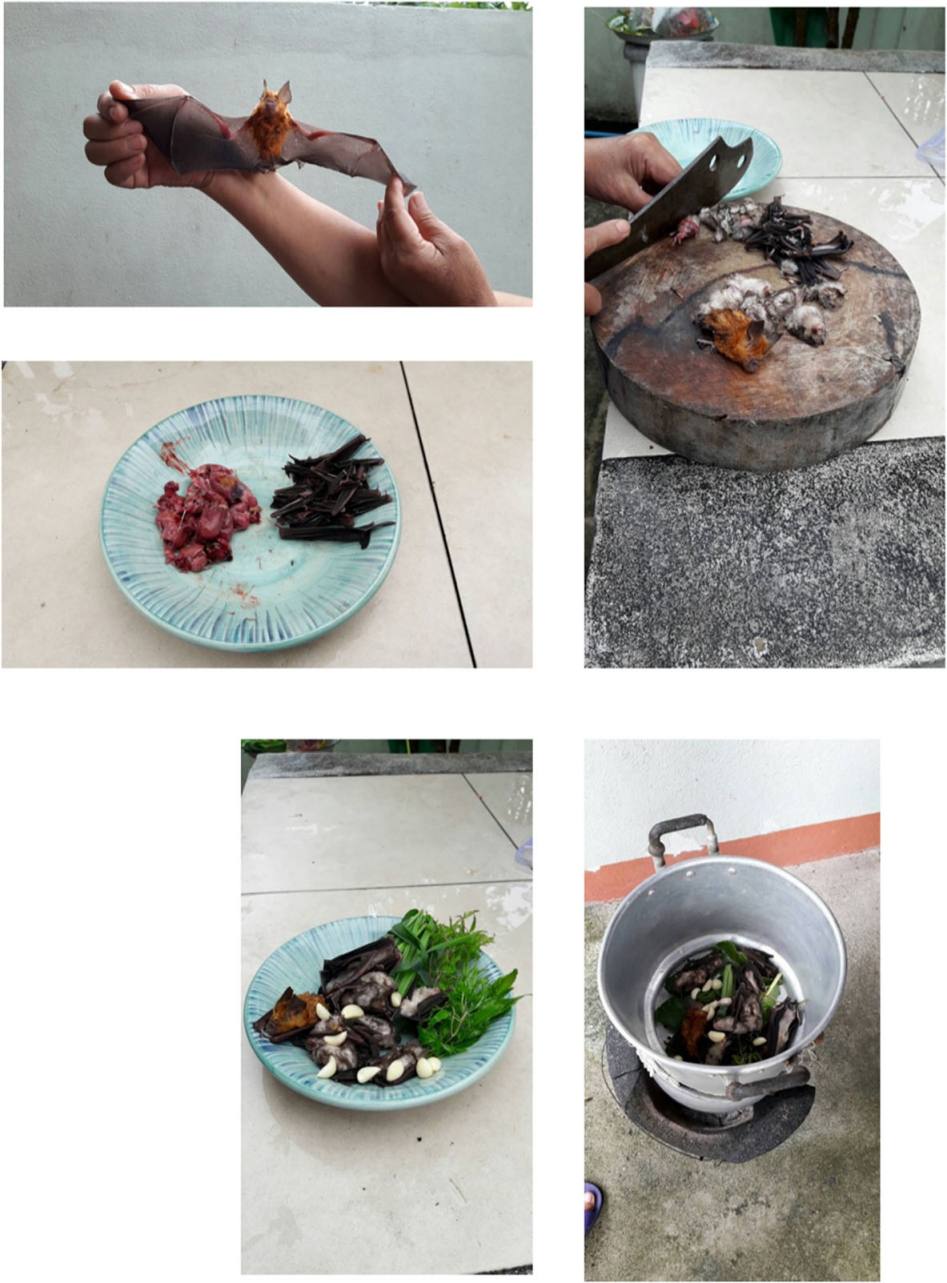
Local food preparation of bat meats (the authors declared the ownership of pictures)

With regard to the details of bat consumption behaviors, we found that 26.04% (25/96) of respondents reported killing bats themselves, followed by vendors (15.63%), hunters (13.54%), and neighbors (11.56%). The participants reported that no children aged 0–10 years old killed bats. With respect to preparing and cooking bats for food, 34.38% (33/96) of respondents prepared bat meat by themselves, followed by spouses (14.58%), neighbors (11.56%), and children (1.04%). Persons reported that the bats were eaten by spouses (48.96%), neighbors (39.58%), and children (13.54%). Most respondents reported eating cooked bat meat (98.96%, 95/96), while only one respondent, a 63-year-old female from Chiang Rai province, reported eating raw bat meat. Bats were obtained by hunting in caves (30.21%), purchasing from local markets (19.79%), hunting by themselves (13.54%), and hunting by neighbors (8.33%). Dishes containing bat were “Kang Om” (spicy vegetable soup) (18.75%), followed by spicy stir fry (13.54%), “Kua Kling” (dry spicy fry with herbs), and deep-fried bat meat (8.33%). The preferred recipes varied among provinces or regions; for example, people in the northern region, Chiang Mai, preferred to cook “Kang Om” while those in southern provinces, Krabi, preferred spicy stir fry to other dishes. The field investigation showed that a typical bat preparation process included butchering the bats, selecting the parts for consumption, adding additional herbs for flavor, and boiling the ingredients in a large pot (Fig. 2).

### Knowledge, attitudes, practices and perceptions regarding bat consumption behaviors

In this study, 23 variables (sociodemographic factors, knowledge, attitudes, practices and perceptions) were involved in the bivariate analysis, and eleven independent variables were eligible for the stepwise logistic regression analysis. These included sociodemographic variables (sex, educational attainment, owning a car, and owning a motorcycle) and variables pertaining to knowledge, attitudes, practices and perceptions (knowledge of one can contract diseases by drinking water from the places where bats live, knowledge of whether it is safe to eat bats, knowledge of whether there are laws pertaining to hunting bats for consumption, practice of allowing their children to play with bats, and attitude of feeling safe in areas where bats live) (Table 5). In the stepwise multiple logistic regression analysis, 5 independent variables were statistically significant (*p* ≤ 0.5). Sex (male) (OR = 2.48, 95% CI 1.49–4.11, *p* < 0.001) and educational attainment (≤ secondary school) (OR = 2.21, 95% CI 1.27–3.85, *p* = 0.005) were statistically significant related to bat consumption behaviors, while knowledge of whether bats are safe to eat (OR = 0.04, 95% CI 0.01–0.25, *p* = 0.001), knowledge of whether there are laws related to hunting bats for consumption (OR = 0.35, 95% CI 0.18–0.71, *p* = 0.004), and allowing children to play with bats (OR = 0.51, 95% CI 0.31–0.81, *p* = 0.006) were statistically significant protective factors against bat consumption behaviors (Table 6).

**Table 5:** Bivariate analysis of bat consumption behavior (Counts (n_2_ = 96), odds ratios, confidence intervals and *p*-values of independent variables)

Variables	Total number (*N* = 626)	Reported bat contact (*n* = 96)		Unadjusted odds ratio	95%Cl	*P*-value
		n	%			
Sociodemographic information						
1. Sex						
*Male*	341	68	70.83	2.29	1.43–3.67	< 0.001*
*Female*	285	28	29.17	1		
2. Age group						
*> 36 years*	132	16	16.67	0.71	0.40–1.27	0.249
≤ *36 years*	494	80	83.37	1		
3. Occupation						
*Agriculture-related occupation*	224	38	39.58	1.21	0.78–1.89	0.399
*Other*	402	58	60.42	1		
4. Marital status						
*Married or cohabiting*	526	82	85.42	1.13	0.62–2.09	0.686
*Single*	100	14	14.58	1		
5. Family size						
*≤ 2 people*	123	23	23.96	1.36	0.81–2.27	0.248
> *2 people*	503	73	76.04	1		
6. Number of children						
*0 children*	195	29	30.21	0.95	0.59–1.52	0.829
> *1 children*	431	67	69.79	1		
7. Educational attainment						
*≤* Secondary school	411	76	79.17	2.24	1.31–3.73	0.002*
Other	215	20	20.83	1		
8. Family monthly income						
≤ *15,000 Baht*	438	64	66.67	0.83	0.53–1.33	0.443
*> 15,000 Baht*	188	32	33.33	1		
9. Own a car						
*Yes*	314	41	42.71	0.70	0.45–1.09	0.113*
*No*	312	55	57.29	1		
10. Own a motorcycle						
*Yes*	574	93	96.87	3.16	0.96–10.35	0.046*
*No*	52	3	3.13	1		
Knowledge, attitudes, practices and perceptions						
11. Human can get diseases from bats						
*True*	40	5	5.21	0.78	0.29 to 2.07	0.607
*False*	586	91	94.79	1		
12. There are no concerns about getting diseases from bats						
*True*	48	8	8.33	1.11	0.50 to 2.55	0.790
*False*	578	88	91.67	1		
13. Bats can transmit diseases to humans						
*True*	84	10	10.42	0.72	0.36 to 1.44	0.348
*False*	542	86	85.58			
14. Bats are economically beneficial to the community						
*Agree*	108	18	18.75	1.13	0.64 to 1.98	0.673
*Disagree*	518	78	81.25	1		
15. One can contract diseases if exposed to bats						
*True*	101	12	12.50	0.71	0.37 to 1.35	0.293
*False*	525	84	87.50	1		
16. One can contract diseases by drinking water from the same place as bats do						
*True*	139	15	15.63	0.61	0.34 to 1.09	0.092*
*False*	487	81	84.37	1		
17. One can contract diseases by eating fruit left by bats						
*True*	170	26	27.08	0.99	0.61 to 1.62	0.986
*False*	456	70	72.92	1		
18. Bat guano is safe to use						
*True*	58	6	6.25	0.61	0.26 to 1.47	0.268
*False*	568	90	93.75			
19. Bats are safe to eat						
*True*	169	1	1.04	0.02	0.00 to 0.16	< 0.001*
*False*	457	95	98.96			
20. There are laws related to hunting bats for consumption						
*True*	196	11	11.46	0.24	0.13 to 0.46	< 0.001*
*False*	430	85	88.54	1		
21. Children are allowed to play with bats						
*True*	431	42	43.75	0.28	0.16 to 0.44	< 0.001*
*False*	195	54	56.25	1		
22. Found dead bats and brought home for food						
*True*	518	75	78.13	0.70	0.41 to 1.19	0.193
*False*	108	21	21.87	1		
23. Feel safe in areas where bats live						
*True*	210	19	19.79	0.44	0.26 to 0.75	0.002*
*False*	416	77	80.21	1		

^*^Cut-off point for stepwise logistic regression at *p* < 0.15

**Table 6:** Stepwise logistic regression analysis of bat consumption behavior (Coefficient, odds ratios, confidence intervals and *p*-values of independent variables)

Variables	Coefficient	Odds ratio	95% CI	*P*-value
Sociodemographic information				
Sex (male)	0.91	2.48	1.49–4.11	< 0.001*
Educational attainment (< secondary school level)	0.79	2.21	1.27–3.85	0.005*
Knowledge, attitudes, practices and perceptions				
Bat are safe to eat (true)	−3.36	0.04	0.01–0.254	0.001*
There are laws related to hunting bats for consumption (agree)	−1.04	0.35	0.18–0.71	0.004*
Children are allowed to play with bats (true)	−0.68	0.51	0.31–0.81	0.006*

*Statistically significant at *p* < 0.05

## Discussion

This is the first quantitative study regarding the risk factors for bat contact and bat consumption behaviors in Thailand. Our study showed that 36.42% of 626 respondents reported bat contact experiences during the past 6 months. The respondents reported finding live bats in houses, the community or tourist locations; eating bats for food; cleaning bat guano from the house or community; finding dead bats in the house; participating in bat guano mining/collecting; cleaning bat carcasses from the house or community; using bat guano as fertilizer; participating in other contact activities; and having been bitten by a bat. This current study showed an incidence of bat contact experiences (36.42%) that was higher than that reported in a study in Canada [30], which showed that 16% of participants had direct contact with bats, and 4% found bats in their houses. However, the frequency of reports of being bitten by bats in the current study was lower (7.02%) than that in the Canadian study (39.00%). Interestingly, bat consumption behavior was shown in the second rank of frequent exposure behavior (42.10%).

From the stepwise logistic regression analysis on bat contact behavior, male, low education attainment and considering bats to be economically beneficial to the community were risk factors for contact with bats. In contrast, farming or agriculture-related occupations were a protective factor against contacting bats, which was different from a study in Guatemala [31]. This might be because other occupations, e.g., temporary workers, had more chances and/or free time to hunt or purchase bats. Our results also showed inappropriate knowledge and attitudes with regard to feeling that it is safe to eat bats, allowing children to play with bats, and feeling safe in areas where bats live, which were influenced by the villagers’ contact with bats. Our observations agreed with the findings from a study in Australia in which respondents had inappropriate perceptions that could lead to more bat contact/exposures [12].

The consumption of wild animals, including bats, a product often called bushmeat, poses challenges for both wildlife conservation and human health [24]. This study showed that 96 (15.34%) of the 626 respondents reported bat consumption during the past 6 months. However, 170 respondents (27.16%) in this study reported eating bats in their lifetime, which was higher than the studies in the Republic of Ghana (23.00%) [22] and Madagascar (25.8%) [23]. Thus, bat consumption incidences in Thailand should be considered a matter for concern.

This study showed that males reported more bat consumption behavior. Our findings were comparable to previous studies in which males were more likely to consume wildlife in Thailand and the Lao PDR [10, 12]. Low education attainment was one of the risk factors that lead to bat contact and consumption. In addition, inappropriate knowledge and attitudes regarding whether it was safe to eat bats, legal to hunt bats, and safe to allow their children to play with bats could also be factors affecting bat consumption. Among the provinces, the respondents from the northern region (Chiang Mai, and Chiang Rai) reported eating bats more than those in other regions. While, the central provinces reported less bat consumption due to their beliefs and social norms per the qualitative study results. Regular law enforcement activities in the central provinces might be one of the contributing factors.

## Conclusions

In conclusion, this study has provided information related to sociodemographic factors, knowledge, attitudes, perceptions, and practices that may influence bat contact and bat consumption behaviors among Thai villagers. The information from this study can be used in the development of communication interventions for zoonotic diseases related to bat contact and bat consumption behaviors in areas with similar environmental and cultural characteristics.

## Supplementary information

**Additional file 1.**

## Data Availability

All data generated or analyzed during this study are included in this published article and supplement tables.

## Abbreviations

CytB: Cytochrome-b
DNP: Department of Natural Parks, Wildlife and Plant Conservation
MNRE: Ministry of Natural Resources and Environment
MOPH: Ministry of Public Health
SRS: Simple random sampling
