# Supplementary material for: Risk factors for bat contact and consumption behaviors in Thailand; a quantitative study
Source: BMC Public Health. 2020 Jun 3;20:841. doi: 10.1186/s12889-020-08968-z (PMC7268181; doi:10.1186/s12889-020-08968-z)
Supplement: Supplementary file 1 — Additional file 1. [file 12889_2020_8968_MOESM1_ESM.docx]

**QUESTIONNAIRE**

**HUMAN-BAT INTERFACE IN THAILAND**

**(HUMAN STUDY – QUANTITATIVE STUDY)**

**INTRODUCTION**

My name is_____(Name of interviewer)__________ and I’m working for ***Center of Excellence for Emerging and Re-emerging Diseases in Animals, Faculty of Veterinary Science, Chulalongkorn University*** to conduct a study survey, entitled ***“Human-Bat Interface in Thailand”***. We’re interviewing people here in your community to learn more about information regarding to bat and human interface in several aspects.

We would very much appreciate your participation in this survey. This questionnaire consists of 5 parts. The questionnaire usually takes between 45 - 60 minutes to complete. Participation in this survey is completely voluntary and you can choose not to answer any individual questions or all of the questions. You may stop participation at any time. However, we hope that you will participate fully in this survey since your views are important.

Whatever information you provide will be kept strictly confidential and will not be shown to persons not associated with this study. Also, no identifying information about you will be kept with the survey responses, including this consent.

Please let me know if anything I have stated is not clear and I will be happy to explain it further to ensure you understand. If you wish to ask questions later, you may contact –

*Prof. Dr. Alongkorn Amonsin*

*Director, Center of Excellence for Emerging and Re-emerging Diseases in Animals, Faculty of Veterinary Science, Chulalongkorn University.*

*Pathumwan, Bangkok 10330*

*Tel: +66 2 218 9771*

*Email address: alongkorn.a@chula.ac.th*

| **Interviewer’s notes** |
| --- |
| ***Location of the household (draw map):***  ***Contact detail for the follow up:*** |

**Section 1: Screening**

| **Questionnaire Code** | **Question** |  | **For Data Entry Staff** |
| --- | --- | --- | --- |
| Q1-01 | Subject Identification Number | **__ __-__ __ __**  **(PROVINCE CODE – ID)**  [For supervisor only, once this respondent is included to the study] |  |
| Q1-02 | Province | [Code ________] |  |
| Q1-03 | District | [Code ________] |  |
| Q1-04 | Sub-district | [Code ________] |  |
| Q1-05 | Area setting | 1. Urban 2. Rural |  |
| Q1-06 | Household number |  |  |
| Q1-07 | Interviewer code | [Code ________] |  |
| Q1-08 | Supervisor code | [Code ________] |  |
| Q1-09 | Date of Interview | ___(dd)/___(mm)/200_ |  |
| Q1-10 | This subject has lived in this area > 6 months | 1. Yes 2. No [STOP |  |
| Q1-11 | Have you exposed to bats in any aspects (e.g. clean bat guano from your household) within 6 months before this interview? | 1. Yes 2. No [STOP] |  |
| Q1-12 | Do you agree for us to draw your blood for laboratory testing? | 1. Yes 2. No [STOP] |  |
| **Q1-13** | **Respondent ID [after the respondent is included into the study)** | **__ __-__ __ __**  **(PROVINCE CODE – ID)** |  |

**Section 2: Socio-demographic Information**

*Note for field researcher: if there are more than 1 eligible respondent in the household, please select only a person who is the leader of the household to be interviewed.*

| **Questionnaire Code** | **Question** |  | **For Data Entry Staff** |
| --- | --- | --- | --- |
| Q2-01 | Respondent sex (observe) | 1. Male 2. Female |  |
| Q2-02 | What is your main occupation? | 1. No occupation/ unemployed 2. Farmer (rice field or other) 3. Hunter 4. Butcher 5. Housewife 6. Shop/ trade (in market) 7. Government officer or employee of government offices 8. Factory worker 9. Student 10. Restaurant worker 11. Daily worker 12. Other, please specify________________________ |  |
| Q2-03 | What is your current marital status? | 1. Single 2. Cohabitating/ Married 3. Divorced/ Separated/ Widowed |  |
| Q2-04 | How many people (including yourself and children) live in this household? | 1. < 2 persons 2. 3-6 persons 3. > 6 persons |  |
| Q2-05 | How many children (<15 years old) live in this household? | Please specify __________________  [Code ________] |  |
| Q2-06 | What is your ethnicity? | 1. Thai (Skip to Q2-08) 2. Non-Thai (Answer Q2-07) |  |
| Q2-07 | If you are not Thai, what is your ethnicity? | Please specify your ethnicity ________________________________  [Code ________] |  |
| Q2-08 | What is the highest level of school you attended? | 1. Never attended school 2. Primary School 3. Secondary School 4. Vocational School 5. Undergraduate 6. Higher than undergraduate |  |
| Q2-09 | Could you please estimate your household income per month (Thai Baht)? | 1. < 15,000 2. 15,001 – 40,000 3. 40,001 – 70,000 4. > 70,001 |  |
| Q2-10 | Your household has a car or truck? | 1. Yes 2. No |  |
| Q2-11 | Your household has a motorcycle? | 1. Yes 2. No |  |

**Section 3: Risk behavior toward focused diseases**

*Instruction for asking questions of this section*

*We are going to discuss on the bat in several aspects. Please feel free to share the information. Your answers are not right or wrong. We would like to learn from you to understand your experience regarding to interaction with bats in your community.*

**Section 3.1 Interaction with bats in houses and community**

| **Questionnaire Code** | **Question** |  | **For Data Entry Staff** |
| --- | --- | --- | --- |
| Q31-01 | Did you or anyone in your household find a dead bat in your household? | 1. Yes 2. No (Skip to Q32-03) |  |
| Q31-02 | If Q31-01 answer Yes, when was that? | 1. > 10 years ago 2. 1.1 – 10 years 3. Last 12 months 4. Last month 5. Last week 6. Within this week |  |
| Q31-03 | As Q31-01, what did you/ your household members do? | 1. Did nothing 2. Brought it home 3. Threw it to trash in the community/ household 4. Burn it 5. Bury it 6. Touch with bared hands and throw it to trash 7. Other, please specify ________________________ |  |
| Q31-04 | Have you/your household members ever found feces of bats in your house? | 1. Yes 2. No (Skip to Q32-07) |  |
| Q31-05 | If Q31-04 answer Yes, when was that? | 1. > 10 years ago 2. 1.1 – 10 years 3. Last 12 months 4. Last month 5. Last week 6. Within this week |  |
| Q31-06 | As Q31-04, what did you/ your household members do? | 1. Did nothing 2. Sweep it 3. Clean it by water 4. Clean it by antiseptics 5. Other, please specify ________________________ |  |
| Q31-07 | Have you/your household members ever found feces of bats in your community? | 1. Yes 2. No (Skip to Q32-10) |  |
| Q31-08 | If Q31-07 answer Yes, when was that? | 1. > 10 years ago 2. 1.1 – 10 years 3. Last 12 months 4. Last month 5. Last week 6. Within this week |  |
| Q31-09 | As Q31-07, what did you/ your household members do? | 1. Did nothing 2. Sweep it 3. Clean it by water 4. Clean it by antiseptics 5. Other, please specify ________________________ |  |
| Q31-10 | Have you/your household members ever found dead bats in your community (but it is not in household e.g. community’s park, or rice field)? | 1. Yes 2. No (Skip to Section 3.2) |  |
| Q31-11 | If Q31-11 answer Yes, when was that? | 1. > 10 years ago 2. 1.1 – 10 years 3. Last 12 months 4. Last month 5. Last week 6. Within this week |  |
| Q31-12 | As Q31-10, what did you/ your household members do? | 1. Did nothing 2. Sweep it 3. Clean it by water 4. Clean it by antiseptics 5. Other, please specify ________________________ |  |
| Q31-13 | If Q31-11 answer Yes, how far of the found area from households in the village? | 1. > 500 meters 2. 501 meters – 1 kilometers 3. 1.1 – 3 kilometers 4. > 3 kilometers |  |

**Section 3.2 Bitten by Bat**

| **Questionnaire Code** | **Question** |  | **For Data Entry Staff** |
| --- | --- | --- | --- |
| Q32-01 | Have you ever bitten by bat in your life? | 1. Yes 2. No (Skip to Q32-04) |  |
| Q32-02 | If Q32-01 answer Yes, when was that? | 1. > 10 years ago 2. 1.1 – 10 years 3. Last 12 months 4. Last month 5. Last week 6. Within this week |  |
| Q32-03 | As Q32-01, what did you do when were bitten by bats? | 1. Do nothing 2. Clean your wound by water immediately 3. Clean your wound by water with soap immediately 4. Clean your wound by water with soap as well as medicine immediately 5. See doctor/ nurse at health facilities 6. Other, please specify ________________________ |  |
| Q32-04 | Have your family member(s) ever bitten by bat in your life? | 1. Yes 2. No (Skip to Section 3.3) |  |
| Q32-05 | As Q32-04, when did your household member (s) get bitten by bats by bats? | 1. > 10 years ago 2. 1.1 – 10 years 3. Last 12 months 4. Last month 5. Last week 6. Within this week |  |
| Q32-06 | As Q32-04, what did your household member (s) do when were bitten by bats? | 1. Do nothing 2. Clean your wound by water immediately 3. Clean your wound by water with soap immediately 4. Clean your wound by water with soap as well as medicine immediately 5. See doctor/ nurse at health facilities 6. Other, please specify ________________________ |  |

**Section 3.3 Bat Consumption**

| **Questionnaire Code** | **Question** |  | **For Data Entry Staff** |
| --- | --- | --- | --- |
| Q33-01 | Have you ever consumed bat in your life? | 1. Yes 2. No (Skip to Q33-12) |  |
| Q33-02 | If Q33-01 answer Yes, when was that? | 1. > 10 years ago 2. 1.1 – 10 years 3. Last 12 months 4. Last month 5. Last week 6. Within this week |  |
| Q33-03 | As Q33-01, who did butcher/ slaughter? | 1. Wife 2. Husband 3. Parents 4. Children (age <10 years old) 5. Neighbor 6. Vendor 7. Hunter 8. Other, specify _____________________________ |  |
| Q33-04 | As Q33-01, who did cook/ prepare it? | 1. Wife 2. Husband 3. Parents 4. Children (age <10 years old) 5. Neighbor 6. Vendor 7. Hunter 8. Other, specify _____________________________ |  |
| Q33-05 | As Q31-01, who did you eat with? (This can be answered more than 1 choices) | 1. Wife 2. Husband 3. Parents 4. Children (age <10 years old) 5. Neighbor 6. Vendor 7. Hunter 8. Other, specify _____________________________ |  |
| Q33-06 | As Q33-01, what was the dish that you ate that made from bats? Please specify the menu, can answer more than 1 dishes: | 1. _______________________________ 2. _______________________________ 3. ________________________________ |  |
| Q33-07 | When was the latest time that you ate bat? | 1. > 10 years ago 2. 1.1 – 10 years 3. Last 12 months 4. Last month 5. Last week 6. Within this week |  |
| Q33-08 | As Q33-07, how did you get the bat? | 1. Catch from caves 2. Catch from forest, but not in caves 3. Catch within community/ village 4. Pick up from carcass in household or in community 5. From hunter 6. From vender in the market 7. From relatives (e.g. parents) 8. From neighbor 9. Other, please specify ______________________________ |  |
| Q33-09 | As Q33-07, did you kill it yourself? | 1. Yes 2. No |  |
| Q33-10 | As Q33-07, who did butcher/ slaughter? | 1. Wife 2. Husband 3. Parents 4. Children (age <10 years old) 5. Neighbor 6. Vendor 7. Hunter 8. Other, specify _____________________________ |  |
| Q33-11 | As Q33-07, who did cook/ prepare it? | 1. Wife 2. Husband 3. Parents 4. Children (age <10 years old) 5. Neighbor 6. Vendor 7. Hunter 8. Other, specify _____________________________ |  |
| Q33-12 | As Q33-07, was the dish raw? | 1. Yes 2. No |  |
| Q33-13 | As Q33-07, what was the dish that you ate that made from bats? Please specify the menu, can answer more than 1 dishes: | 1. __________________________ 2. __________________________ 3. __________________________ |  |
| Q33-14 | Have you ever found bats selling in your community? | 1. Yes 2. No (Skip to Section 4) |  |
| Q33-15 | As Q33-14, where was it selling? | 1. Local market within the 2. None, it was sharing within family members 3. Hunter 4. Neighbor 5. Other, please specify _________________________ |  |
| Q33-16 | As Q33-14, did you or your household members buy it? | 1. Yes (Answer Q33-15 and Q33-16) 2. No (Answer Q33-17) |  |
| Q33-15 | As Q33-14, when did you or your household members buy it? | 1. > 10 years ago 2. 1.1 – 10 years 3. Last 12 months 4. Last month 5. Last week 6. Within this week |  |
| Q33-16 | As Q33-15 answer Yes, why was that? | Please specify__________________________ |  |
| Q33-17 | As Q33-15 answer No, why was that? | Please specify__________________________ |  |

**Section 4: knowledge, attitude, practice, and Perception toward bats**

***Instruction for asking questions of this section***

*Now, I’m going to read you some statements about bats. Please tell me if you feel –*

1. *Totally agree*
2. *Somewhat agree*
3. *Neutral*
4. *Somewhat disagree*
5. *Totally disagree*
6. *Don’t know*
7. *Not response (if respondent didn’t answer any)*

| **Questionnaire Code** | **Question** | **Interviewer fills the answer**  **(as number)** | **For Data Entry Staff** |
| --- | --- | --- | --- |
| Q4-01 | People can get diseases from bats. |  |  |
| Q4-02 | I’m not concerned about disease that people can get from bats. |  |  |
| Q4-03 | Bats are not harmful animals. |  |  |
| Q4-04 | Bats are important for economic status in this community. |  |  |
| Q4-05 | I can get diseases from bats if I touch them. |  |  |
| Q4-06 | People can get diseases from bat by sharing drinking water with them. |  |  |
| Q4-07 | People can’t get diseases from bat by eating fruit that might be bitten by bats. |  |  |
| Q4-08 | Bat guano is safe to use as fertilizer. |  |  |
| Q4-09 | It is fine to consume bats. |  |  |
| Q4-10 | If there is no law enforcement, I will catch bats in the forest for consumption. |  |  |
| Q4-11 | I allow my child/ren to touch bats. |  |  |
| Q4-12 | When I found a dead bat, I will bring it home and cook as food. |  |  |
| Q4-13 | I feel safe to enter to area that have bats. |  |  |

**Section 5: Communication regarding to bats**

| **Questionnaire Code** | **Question** |  | **For Data Entry Staff** |
| --- | --- | --- | --- |
| Q5-01 | Which communication channel that you use the most in the past 12 months for news? | 1. Newspaper 2. Radio 3. Television 4. Social media 5. Family members 6. Health personnel 7. Village health volunteer 8. Neighbor 9. Teacher 10. Monk 11. Nothing 12. Other, please specify________________________ |  |
| Q5-02 | Who is the most trust information regarding to diseases or disease prevention or disease outbreak? | 1. Newspaper 2. Radio 3. Television 4. Social media 5. Family members 6. Health personnel 7. Village health volunteer 8. Neighbor 9. Teacher 10. Monk 11. Nothing 12. Other, please specify________________________ |  |
| Q5-03 | Once you get sick, which person or places that you will visit/ talk with first (aside from your family members? | 1. Health personnel in local health center 2. Medical doctor/ nurse in hospital 3. Village health volunteers 4. Neighbor 5. Teacher 6. Monk 7. No one 8. Other, please specify________________________ |  |
| Q5-04 | What diseases did you have heard? | Please specify________________________ |  |
| Q5-05 | What bat-borne diseases did you have heard? | Please specify________________________ |  |
| Q5-06 | What diseases that you got sick? | Please specify________________________ |  |
| Q5-07 | What bat-borne diseases that you got sick? | Please specify________________________ |  |
| Q5-08 | What diseases that your family members got sick? | Please specify________________________ |  |
| Q5-09 | What bat-borne diseases that family members got sick? | Please specify________________________ |  |

**Section 6: Laboratory testing information**

| **Questionnaire Code** | **Question** |  | **For Data Entry Staff** |
| --- | --- | --- | --- |
| Q6-01 | Do you agree for us to draw your blood for laboratory testing? | 1. Yes 2. No |  |
| Q6-02 | Results of laboratory testing  Results in attachment No. ___ |  |  |
